# A Pathological Study of Acute Pulmonary Toxicity Induced by Inhaled Kanto Loam Powder

**DOI:** 10.3390/ijms19020416

**Published:** 2018-01-31

**Authors:** Yoshimi Kobayashi, Akinori Shimada, Takehito Morita, Kenichiro Inoue, Hirohisa Takano

**Affiliations:** 1 Department of Veterinary Pathology, Tottori University, 4-101 Koyama Minami, Tottori-shi, Tottori 680-8553, Japan; guildmiyoshi@yahoo.co.jp (Y.K.); morita@muses.tottori-u.ac.jp (T.M.); 2 Laboratory of Pathology, School of Life and Environmental Science, Azabu University, 1-17-71 Fuchinobe, Sagamihara-shi, Kanagawa 252-5201, Japan; 3 School of Nursing, University of Shizuoka, Shizuoka-shi, Shizuoka 422-8526, Japan; inoue-k@u-shizuoka-ken.ac.jp; 4 Department of Environmental Engineering, Kyoto University Graduate School of Engineering, Kyoto-shi, Kyoto 615-8530, Japan; takano.hirohisa.4x@kyoto-u.ac.jp

**Keywords:** Asian sand dust, inhalation, Kanto loam powder, pulmonary toxicity

## Abstract

The frequency and volume of Asian sand dust (ASD) (Kosa) are increasing in Japan, and it has been reported that ASD may cause adverse respiratory effects. The pulmonary toxicity of ASD has been previously analyzed in mice exposed to ASD particles by intratracheal instillation. To study the pulmonary toxicity induced by inhalation of ASD, ICR mice were exposed by inhalation to 50 or 200 mg/m^3^ Kanto loam powder, which resembles ASD in elemental composition and particle size, for 6 h a day over 1, 3, 6, 9, or 15 consecutive days. Histological examination revealed that Kanto loam powder induced acute inflammation in the whole lung at all the time points examined. The lesions were characterized by infiltration of neutrophils and macrophages. The intensity of the inflammatory changes in the lung and number of neutrophils in both histological lesions and bronchoalveolar lavage fluid (BALF) appeared to increase over time. Immunohistochemical staining showed interleukin (IL)-6- and tumor necrosis factor (TNF)-α-positive macrophages and a decrease in laminin positivity in the inflammatory lesions of the lung tissues. Electron microscopy revealed vacuolar degeneration in the alveolar epithelial cells close to the Kanto loam particles. The nitric oxide level in the BALF increased over time. These results suggest that inhaled Kanto loam powder may induce diffuse and acute pulmonary inflammation, which is associated with increased expression of inflammatory cytokines and oxidative stress.

## 1. Introduction

Asian sand dust (ASD) (known as Kosa aerosol) originating from the arid deserts of Mongolia and China, causes severe air pollution annually in the Asia-Pacific area, including China, Korea, and Japan [1,2]. Epidemiological studies of ASD show that human exposure to ambient ASD particles is associated with an increase in pulmonary and cardiovascular problems and an increase in daily mortality in Korea [3,4], Taiwan [5], and Japan [6,7]. The frequency and scale of dust events giving rise to ASD aerosols have increased rapidly in the east Asian region since 2000 [8]. Due to recent environmental changes, humans are at an increased risk of exposure to ASD and the resultant adverse health effects on the respiratory system [9].

A previous study [10] reported that the major mineralogical component of ASD is silica (SiO_2_). Occupational exposure to crystalline silica is associated with silicosis, lung cancer, pulmonary tuberculosis, and chronic obstructive pulmonary disease [11]. Moreover, silicosis patients often develop autoimmune diseases [12,13,14]. It has been reported that experimental chronic intratracheal exposure to crystalline silica causes granuloma formation and/or fibrosis in rats [15,16,17,18]. In addition, granulomatous inflammation, which is characterized by the accumulation of epithelioid macrophages containing crystalline silica particles, has been reported in the pulmonary lymph nodes of rats exposed by inhalation to crystalline silica [19,20]; lymph node silicosis was also reported in miners with prolonged silica exposure [21].

Intratracheal instillation of high doses of ASD produced acute [22,23] and chronic [24,25] inflammatory changes in the lung tissues. The chronic changes were similar to those caused by silica exposure and consisted of a focal infiltration of lymphocytes with an accumulation of epithelioid macrophages, granuloma formation in the lung, and aggregation of particle-containing macrophages in the pulmonary lymph nodes at two and three months after instillation. These findings suggest that precautions for the prevention of ASD exposure are required for the protection of public health and that experimental studies of ASD toxicity after inhalation are necessary.

The purpose of this study was to describe the acute lung toxicity induced by the inhalation of Kanto loam powder, which resembles ASD in elemental composition and particle size, in mice.

## 2. Results

### 2.1. Gross Findings in the Lungs

There were no gross changes in the lungs at 1, 3, 6, 9, or 15 days after exposure to either 50 or 200 mg/m^3^ of Kanto loam powder.

### 2.2. Histopathology of the Lungs

**Figure 1:**
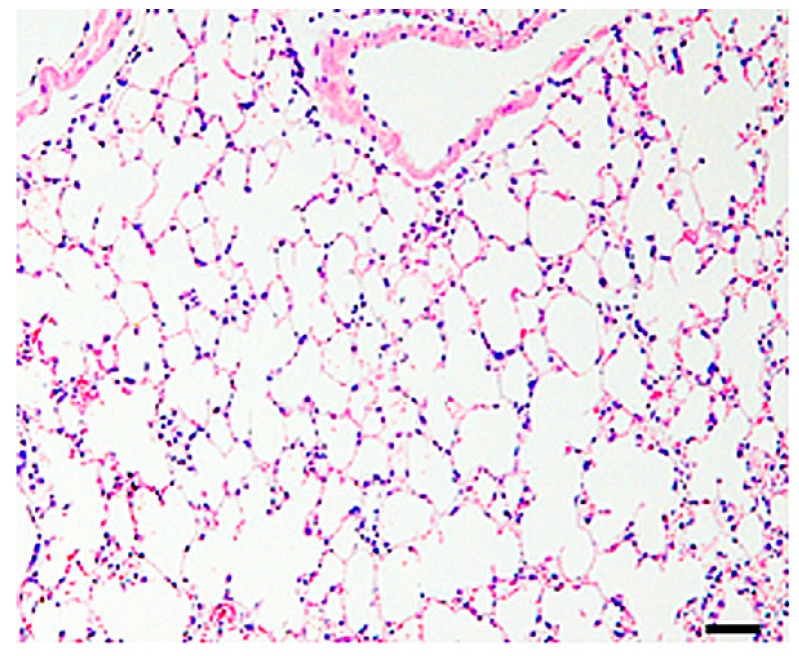
Histological findings of the control lungs, showing no changes. HE. Bar = 50 µm.

Lungs from control mice showed no pathological changes (Figure 1).

**Figure 2:**
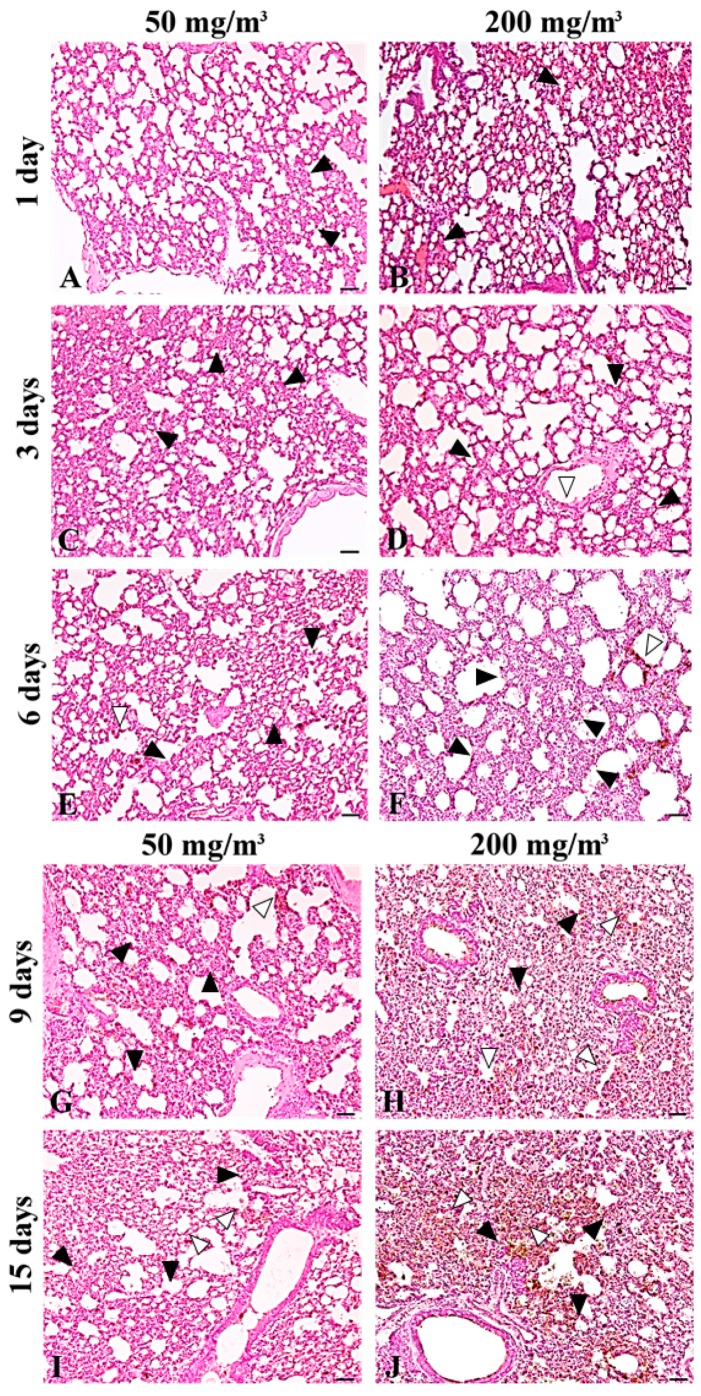
Histological examination of the lung taken from mice treated with Kanto loam powder showed acute inflammatory changes. Representative lung sections taken from mice treated with 50 mg/m^3^ of the powder at one day (**A**); three days (**C**); six days (**E**); nine days (**G**); and 15 days (**I**) after exposure, and from mice treated with 200 mg/m^3^ Kanto loam powder at one day (**B**); three days (**D**); six days (**F**); nine days (**H**); and 15 days (**J**) after exposure. Diffuse deposition of the particles (white arrowheads) at the bronchial mucosa and alveolar wall, and infiltration of inflammatory cells (neutrophils and macrophages) (black arrowheads) in the alveolar wall and alveolar space are observed; these changes become prominent with both time and concentration of the particles. HE. Bars = 50 µm.

**Figure 3:**
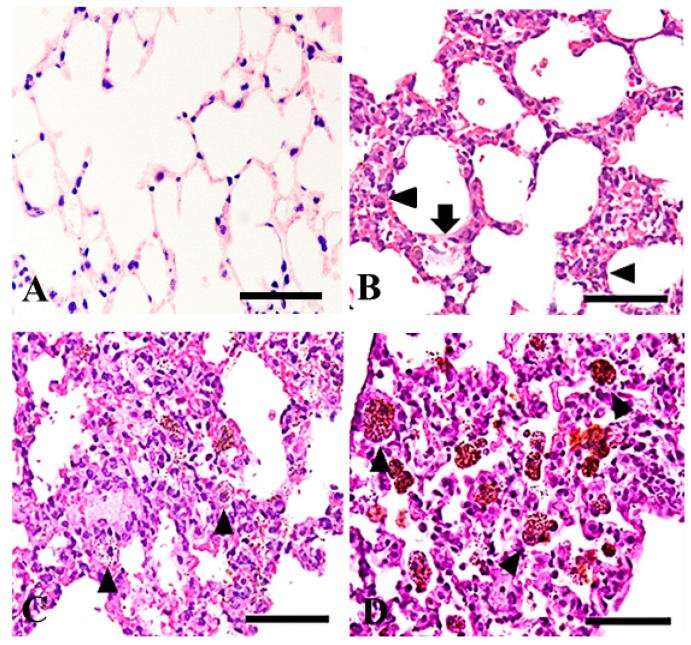
Higher power magnification of the histological findings of the control lungs (**A**); of mice treated with 50 mg/m^3^ of the powder for one day (**B**); treated for 15 days (**C**); of mice treated with 200 mg/m^3^ of the powder for 15 days (**D**). Infiltration of neutrophils into alveoli and increased number of macrophages (black arrowheads), and exudation of serum protein in the alveolar space (black arrow) (**B**). Macrophages with increased cytoplasm containing the particles are shown (black arrow heads) (**C**,**D**). HE. Bars = 50 µm.

In the lungs from all of the particle-treated mice, diffuse deposition of the particles and acute inflammatory changes were observed, and the intensity of the inflammatory changes appeared to increase over time and with increasing particle concentration (Figure 2A–J). We observed infiltration of neutrophils into the alveolar space (Figure 3B), an increase in the number of macrophages containing cytoplasmic particles (Figure 3B), and intra-alveolar hemorrhage and the exudation of serum protein into the alveolar spaces (Figure 3B). Macrophages with increased cytoplasm were observed in the lungs of the mice treated with 50 mg/m^3^ of the particles for 15 days (Figure 3C) and in the lungs of the mice treated with 200 mg/m^3^ of the particles for 6, 9, and 15 days (Figure 3D).

### 2.3. Histopathology of the Pulmonary Lymph Nodes

Pulmonary lymph nodes from control mice showed no pathological changes.

**Figure 4:**
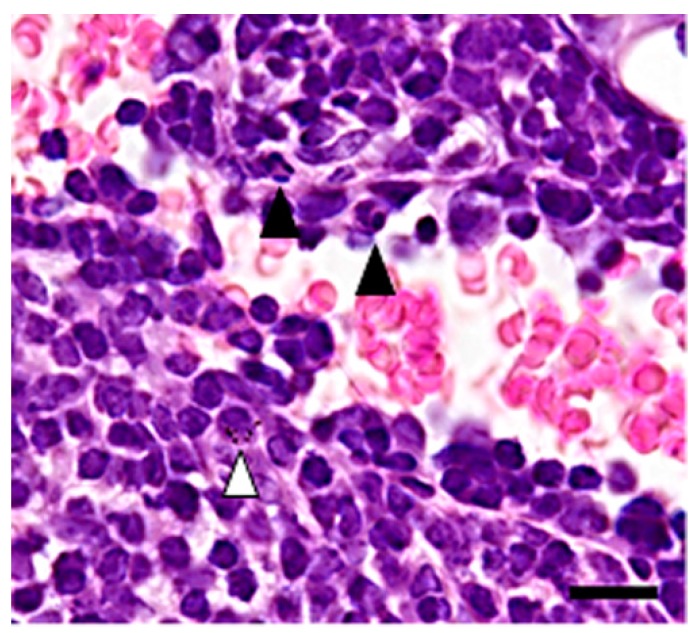
Histological changes of the lymph nodes taken from mice treated with 50 mg/m^3^ of the powder for one day. Increased number of neutrophils (black arrowheads) and a macrophage containing the particles in the sinus (a white arrowhead). HE. Bar = 50 µm.

In the pulmonary lymph nodes taken from all of the particle-treated mice, an increased number of particle-containing neutrophils and macrophages were observed in the lymphatic sinus (Figure 4).

### 2.4. Immunohistochemistry of the Lung

#### 2.4.1. α-SMA

**Figure 5:**
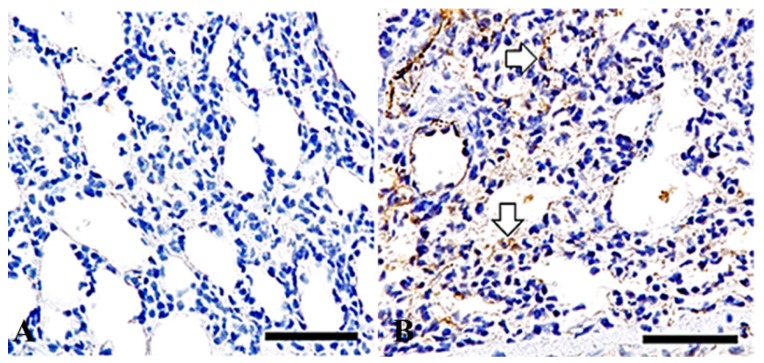
Alpha-SMA immunohistochemistory in the lungs of mice treated with 50 mg/m^3^ of the powder for one day (**A**) and treated for 15 days (**B**). Spindle-shaped to elongated cells in the inflammatory lesions show positive immunolabelings for α-smooth muscle actin ((**B**), white arrows). Bars = 50 µm.

Alpha-SMA-positive spindle-shaped cells were observed in the lungs of the mice treated with 50 mg/m^3^ of the particles at 15 days after exposure, exclusively (Figure 5B).

#### 2.4.2. Laminin

**Figure 6:**
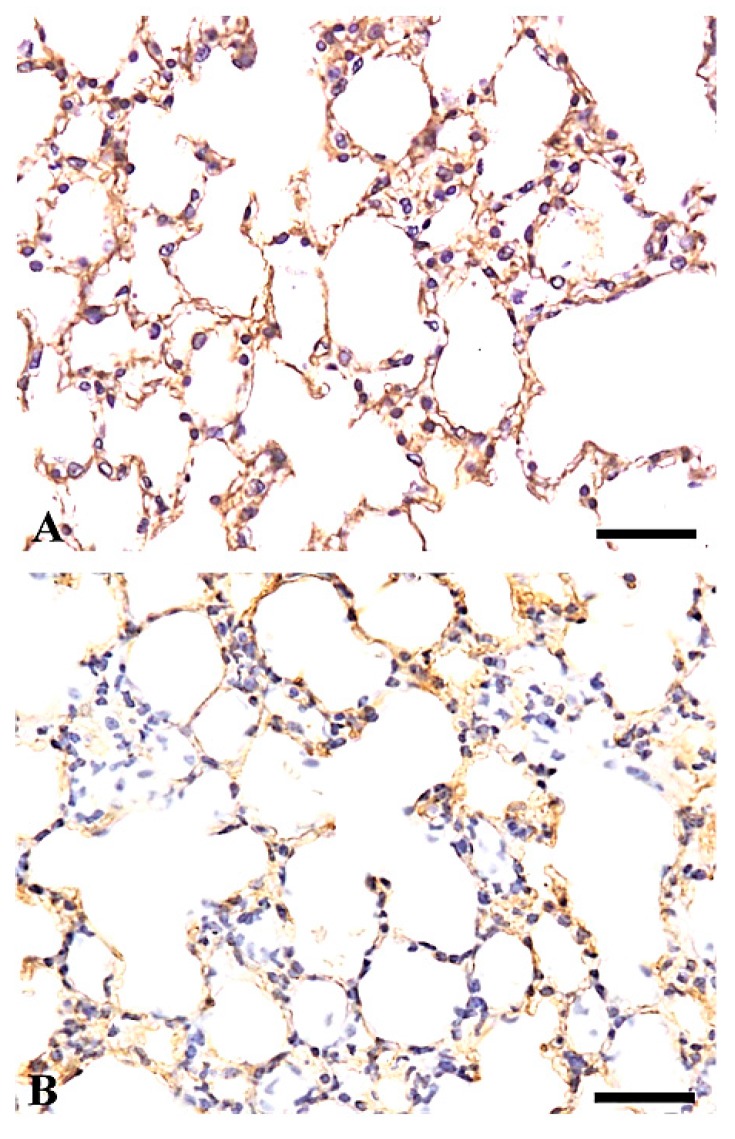
Laminin immunohistochemistry in the lungs of control mice (**A**) and of mice treated with 50 mg/m^3^ of the powder for one day (**B**). Positive, continuous, distinct thin lines are shown in the basement membrane of the alveoli (**A**). Weakly positive discontinuous staining is shown in the basement membrane of the alveoli in the inflammatory lesions (**B**). Bars = 30 µm.

In the control lung tissue, laminin staining revealed continuous, distinct thin lines in the basement membranes of the bronchioles, alveolar walls, and blood vessels (Figure 6A). Reduced laminin positivity was observed in the basement membranes of the alveolar walls in the inflammatory lesions in the lungs of the particle-treated mice (Figure 6B).

#### 2.4.3. TNF-α, IL-6, and iNOS

**Figure 7:**
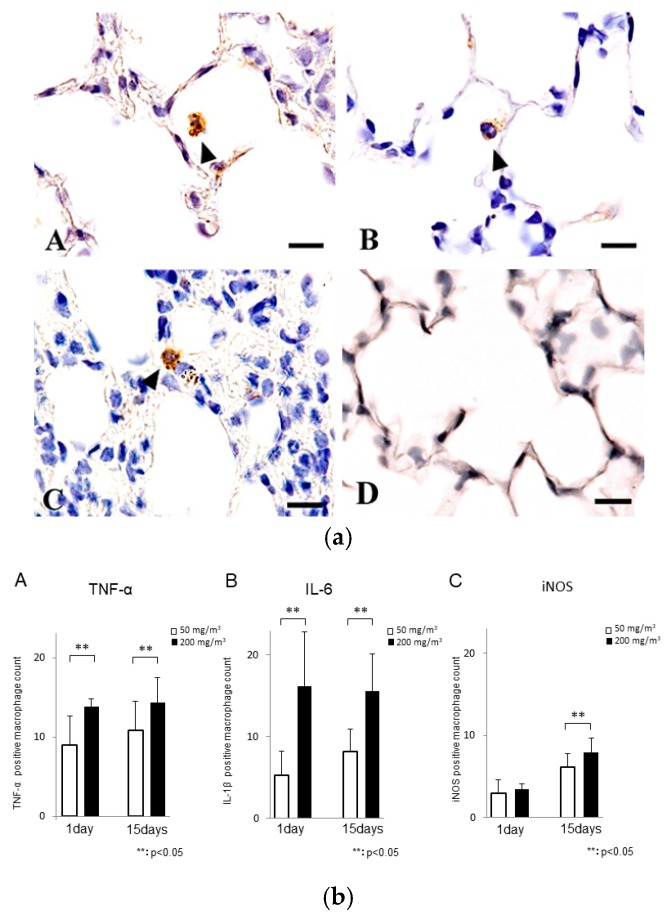
(**a**) TNF-α (A), IL-6 (B), and iNOS (C) immunohistochemistry of the lungs of mice treated with 50 mg/m^3^ of the powder for one day. Macrophages containing the brown particles show positive immunoreactivity for TNF-α, IL-6, and iNOS (black arrowheads). Control (lungs of mice with no treatment, TNF-α) (D). Bars = 10 µm. (**b**) A semi-quantative analysis of the immunohistochemical reactions (A: TNF-α, B: IL-6, C: iNOS) in the lungs of mice treated with 50 mg/m^3^ (open bars) and 200 mg/m^3^ (filled bars) of the powder for one day and for 15 days (** *p* < 0.05).

The cytoplasm of macrophages, which were lysozyme-positive [26], in the inflammatory lesions of the particle-treated mice stained positively for TNF-α, IL-6 and iNOS (Figure 7a). Semi-quantitative analysis revealed a larger number of TNF-α, IL-6, and iNOS positive macrophages in the lungs of mice treated with 200 mg/m^3^ of the powder (Figure 7b).

### 2.5. Electron Microscopy Analysis

**Figure 8:**
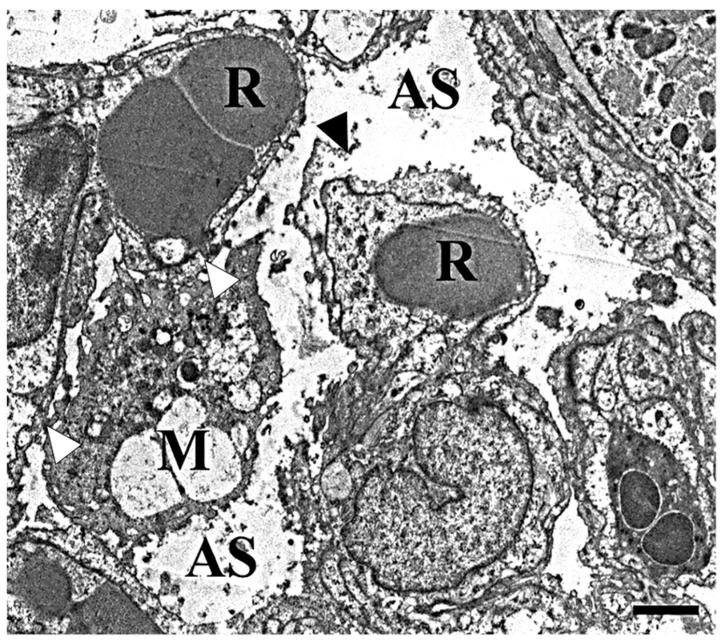
Transmission electron microscopic images of the inflammatory lesions in the lung from mice treated with 50 mg/m^3^ of the powder for three days. Destruction of the alveolar walls (black arrowhead) with epithelial cells showing vacuolar degeneration was observed in the inflammatory lesions. Note that the macrophage containing the particles is closely located to the alveolar wall (white arrowheads). AS: alveolar space, R: red blood cell, M: macrophage. Bar = 1 µm.

In the lungs of the mice treated with 50 mg/m^3^ of the powder for three days, destruction of the alveolar walls and epithelial cells exhibiting vacuolar degeneration were observed in the inflammatory lesions (Figure 8).

### 2.6. Cytology and Measurement of NO in the BALF

**Figure 9:**
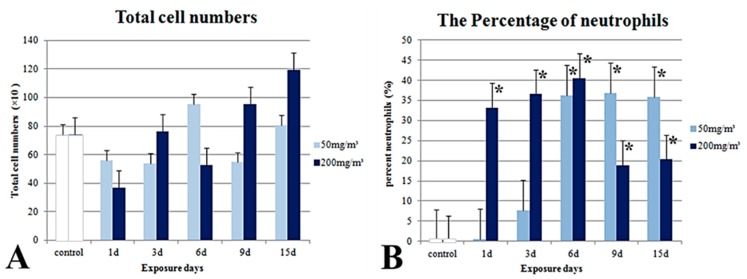

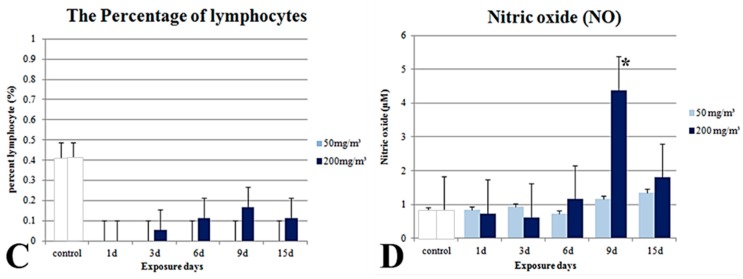
Cellular and biochemical parameters of the bronchoalveolar lavage fluid. Total cell numbers (**A**); The percentage of neutrophils (**B**); The percentage of lymphocytes (**C**); Nitric oxide (D). d: day, * significantly different from the control group, *p* < 0.05.

The cytology results and NO measurements in the BALF are described in Figure 9. Significant changes in the BALF total cell numbers were not observed in the mice from any group, including the control group (Figure 9A). However, an increase in the number of BALF neutrophils was observed after the treatment (Figure 9). The percentage of neutrophils appeared to increase with the increasing number of exposure days after treatment in both the groups treated with 50 mg/m^3^ and 200 mg/m^3^ of the powder (Figure 9B). The percentage of neutrophils increased steeply to 40% at six days after the treatment, and then decreased to 20% at nine days and 15 days after the treatment (Figure 9B). The percentage of lymphocytes was not significantly altered after the treatment at any time point (Figure 9C). A significant increase in the BALF NO level was observed in the mice treated with 200 mg/m^3^ of the powder for nine days (Figure 9D).

## 3. Discussion

Acute pulmonary toxicity caused by inhalation exposure of Kanto loam powder, which resembles ASD in elemental composition and particle size, was pathologically examined in this study over a period of 1 to 15 days after exposure. In the exposed groups, infiltration of inflammatory cells, dominated by neutrophils and macrophages, was observed in both lung tissues and BALF. Histological sections of the lungs taken from the mice treated with 50 mg/m^3^ and 200 mg/m^3^ of the particles showed acute alveolitis consisting of an increased number of particle-containing neutrophils and macrophages, intra-alveolar hemorrhage, exudation of serum protein into the alveolar spaces, and diffuse deposition of particles at the bronchi and in alveolar epithelial cells. The intensity of the inflammatory changes in the lung and the number of neutrophils in the BALF from particle-treated mice appeared to increase with both time after exposure and particle concentration. Acute [22,23] and chronic [24,25] pulmonary toxicity induced by intratracheally-instilled ASD is characterized by purulent inflammation, thickening of alveolar walls, fibrosis, and formation of granulomas. Similar lesions were also reported after experimental inhalation of crystalline silica [16,17,18,27,28].

In the normal lung tissues, type 1 alveolar epithelial cells and vascular endothelial cells are closely packed together across the basement membrane [29]. In this study, the destruction of the alveolar walls in the inflammatory lesions was observed by electron microscopy. The degeneration and detachment of type 1 alveolar epithelial cells, and the dissociation of the basement membrane between type 1 alveolar cells and endothelial cells were observed in the pulmonary lesions of the mice treated with colloidal silica [30]. These findings suggest that silicon dioxide (SiO_2_), which is the major component of Kanto loam powder, might be, in part, responsible for pulmonary lesion formation.

Laminin, which is present in the pulmonary basement membranes and is used as a marker for normal alveolar structures [31], plays a central role in the stability of basement membranes, as well as in the control of cellular interactions. In this study, inflammatory lesions in the lung tissues showed a decrease in laminin immunopositivity resulting from the destruction of the pulmonary tissues, including the basement membrane. Alpha-SMA is a marker for smooth muscle fibers, as well as myofibroblasts. Myofibroblasts have a role in the repair of injured tissue by producing connective tissue components during the chronic phase of inflammation [32,33]. The increase in α-SMA immunopositivity observed in the lung inflammatory lesions in the mice treated with the Kanto loam powder may have been produced in an attempt to repair the tissue.

TNF-α plays important roles in acute inflammation, such as activation of inflammatory cells and induction of secondary tissue injury. The expression of chemokines is modulated by the presence of TNF-α [34]. TNF-α is produced by activated macrophages, lymphocytes, and endothelial cells, as well as other various cell types [35]. IL-6, which has multiple functions, is produced by many cell types, such as T lymphocytes, monocytes, endothelial cells, and fibroblasts [36]. The release of inflammatory cytokines, such as TNF-α and IL-6, in pulmonary lesions was observed in animals treated with ASD [22,37], quartz [38], and crystalline silica [39]. In this study, particle-containing macrophages in the inflammatory lesions also stained positively for TNF-α and IL-6. Inflammatory cytokines, including TNF-α and IL-6, may be involved in the development of the inflammatory lesions observed in this study.

NO and reactive oxygen species are reported to cause severe oxidative stress [17]. In the BALF from rats instilled with crystalline silica, messenger RNA levels of iNOS, an inducible enzyme that produces NO, increased [40,41]. The activation of iNOS in the inflammatory cells and the production of NO were reported to be associated with silica-induced damage of the lung [20,42]. In this study, particle-containing macrophages were positive for iNOS immunoreactivity. A significant increase in BALF NO levels was observed in the mice treated with 200 mg/m^3^ of the powder for nine days. These findings suggest that oxidative stress may be, in part, responsible for the development of acute lung toxicity induced by Kanto loam powder.

## 4. Materials and Methods

### 4.1. Animals

A total of 66 male five-week-old ICR mice were obtained from CLEA JAPAN Inc. (Tokyo, Japan). The animals were fed a diet, CE-2, purchased from CLEA JAPAN and water was given ad libitum. The mouse cages were placed in a conventional room, where the temperature was maintained at approximately 25 °C and the humidity was maintained at 55 to 70%. All animal experiments were performed according to the Tottori University Guidelines for Animal Welfare (12－T―21, 2 July 2012 http://grc1.med.tottori-u.ac.jp/files/12337.pdf?_=20141007.pdf). Body weight changes were recorded weekly to assess the general health status of the mice.

### 4.2. Materials

The mice were exposed to Kanto loam powder (JIS Z 8901, class 11 ultrafine), obtained from the Association of Powder Process Industry and Engineering (Kyoto, Japan). Kanto loam powder consists of the following: 34–40% SiO_2_, 26–32% Al_2_O_3_, 0–7% MgO, 17–23% Fe_2_O_3_, and 0–3% CaO, with 0–4% ignition loss. The mean diameter of the particles is approximately 1.6–2.3 µm according to the manufacturer’s data sheet. ASD particles (CJ-2) contain 28.0% Si, 5.9% Al, 5.3% Ca, 3.0% Fe, 1.7% K, and 1.6% Mg [23,24,25], and the mean diameter of the 2010 and 2011 ASD, transported in Korea over long distances, were reported to be 2.5 and 2.9 µm, respectively [43]; Kanto loam powder resembles ASD in elemental composition and particle size.

### 4.3. Study Protocol

A total of 66 mice were divided into a control group (*n* = 6) and five exposure groups (*n* = 12) as follows: one-day exposure group, three-day exposure group, six-day exposure group, nine-day exposure group, and 15-day exposure group. Each exposure group was divided into two subgroups according to the concentration of Kanto loam powder: a 50 mg/m^3^ group and a 200 mg/m^3^ group [17,26,27].

**Figure 10:**
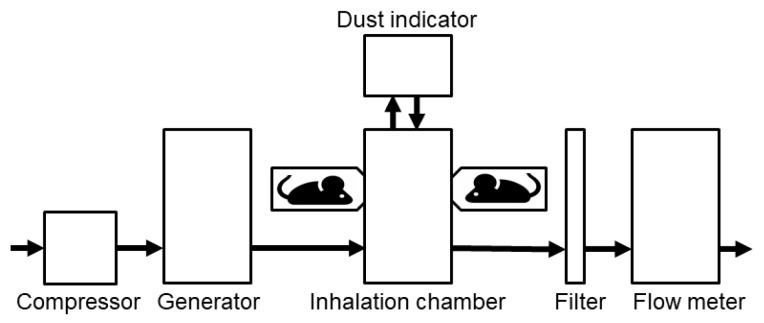
Diagram of the nose inhalation system.

Six mice were placed in an acrylic holder that was attached to the nose-only inhalation exposure apparatus (SIS-C, SIBATA, Soka, Japan) and exposed to Kanto loam powder at concentrations of 50 or 200 mg/m^3^ for 6 h a day over 1, 3, 6, 9, or 15 consecutive days (Figure 10). The test material was generated using a dust feeder (DF-3, SIBATA). The control mice were exposed to clean air.

After exposure, the animals were deeply anesthetized by intraperitoneal injection of sodium pentobarbital and killed by exsanguination.

### 4.4. Pathological Examination

A pathological examination was performed on three mice from each group. Portions of the lungs and pulmonary lymph node were fixed by immersion in 10% neutral-buffered formalin for one day. The formalin-fixed tissues of the lungs and pulmonary lymph node were routinely processed, and embedded in paraffin for histopathological and immunohistochemical examinations. Approximately 3-µm-thick sections were cut and stained with hematoxylin and eosin (HE). The pathological examination was performed by two pathologists.

### 4.5. Immunohistochemistry

Paraffin-embedded lung sections from the mice exposed to 50 mg/m^3^ of the Kanto loam powder or control mice exposed to clean air were used for immunohistochemical detection of alpha smooth muscle actin (α-SMA), laminin, lysozyme, tumor necrosis factor alpha (TNF-α), interleukin-6 (IL-6), and inducible nitric oxide synthase (iNOS) levels. Antigen retrieval was performed on the sections by incubating in citrate buffer solution (pH = 5.4) and microwaving (α-SMA, IL-6, TNF-α, and iNOS) or by treating with protein kinase (laminin, lysozyme). Endogenous peroxidase activity was quenched by immersion in 3% H_2_O_2_ at room temperature for 30 min. The slides were then blocked with 10% normal goat serum for 5 min with microwave treatment. Thereafter, the sections were incubated with the primary antibodies overnight at 4 °C (anti-α-smooth muscle actin, Dako, Glostrup, Denmark, 1:80 dilution; anti-laminin, Dako, 1:2000 dilution; anti-lysozyme, Dako, 1:500 dilution; anti-TNF-α, Monosan, Uden, The Netherlands, 1:15 dilution; anti-IL-6, Abcam, Tokyo, Japan, 1:200 dilution; and anti-iNOS, Abcam, Cambridge, UK, 1:100 dilution). The primary antibodies were replaced with phosphate-buffered saline in the negative controls. After incubation with the primary antibodies, peroxidase-labeled polymers conjugated to secondary anti-mouse and anti-rabbit antibodies (En Vision + kit/HRP (DAB), Dako) were applied to the sections for 30 min at room temperature. The sections were stained with the 3,3′-diaminobenzidine tetrahydrochloride (DAB) as a chromogen, and counterstained with hematoxylin. Positive reactions are indicated by brown staining. In the lungs of mice treated with 50 mg/m^3^ and 200 mg/m^3^ of the powder for one day and for 15 days, a semi-quantitative evaluation of the immunohistochemical reactions (TNF-α, IL-6, iNOS) was conducted by counting the number of stained cells in 10 microscopic fields at 400×. This was performed five times and the mean values were calculated and recorded.

### 4.6. Electron Microscopy

The lung accessory lobes of the mice treated with 50 mg/m^3^ of the Kanto loam powder were fixed in 10% neutral phosphate-buffered formalin and examined by electron microscopy. The tissues were trimmed to 1 mm × 1 mm × 1 mm and post-fixed in 1% osmium acid fixative. After osmification, the tissues were rinsed in 0.1 M phosphate buffer, dehydrated through a graded ethanol series, transitioned through n-butyl glycidyl ether (QY-1) (Nisshin EM Co., Ltd., Tokyo, Japan), and infiltrated and embedded in Quetol-812 epoxy formulation (Nisshin EM Co., Ltd., Tokyo, Japan). Afterward, thick sections were cut, mounted on glass slides, stained with toluidine blue, and examined by light microscopy. Thin sections were mounted on 200-mesh copper fine grids (Nisshin EM Co., Ltd., Tokyo, Japan), and stained with uranyl acetate, followed by post-staining in lead acetate. The sections were then examined using a SU8020 field emission-type scanning electron microscope at 25 kV accelerating voltage (Hitachi Ltd., Tokyo, Japan).

### 4.7. Analysis of Bronchoalveolar Lavage Fluid (BALF)

The BALF from three animals of each group was examined. After euthanasia, the trachea was cannulated, and the lungs were lavaged with three injections of 1.2 mL saline maintained at 37 °C. The lavage fluid was harvested by gentle aspiration. The three lavage samples were combined into a plastic tube, cooled to 4 °C, and centrifuged at 3000 rpm for 10 min. The supernatants were stored at −80 °C until the nitric oxide (NO) analysis was performed. NO was analyzed using a Griess Reagent System (Promega Corporation, Madison, WI, USA) according to the manufacturer’s protocol. The total number of cells, cell viability, and differential cell counts were analyzed using the cell pellets. Cell viability was assessed by trypan blue dye exclusion using a hemocytometer chamber. The differential cell counts were assessed on cytology slide preparations. The slides were stained with Diff-Quick (International Regents Corp., Kobe, Japan), and a total of 200 cells were counted using a light microscope.

### 4.8. Statistical Analysis

All data were expressed as mean ± standard deviation. Statistical analysis of the cellular and biochemical parameters of BALF and immunohistopathological changes was performed using Student’s *t*-test for two-group comparisons. For all comparisons, *p* values less than 0.05 were considered statistically significant.

## 5. Conclusions

This study demonstrated that inhalation exposure of 50 mg/m^3^ or 200 mg/m^3^ of Kanto loam powder, which resembles ASD in elemental composition and particle size, caused acute and diffuse inflammatory changes in the lung. The direct effect of the Kanto loam powder and the indirect effects produced by the secondary release of inflammatory cytokines and NO may be responsible for these changes. However, an inhalation exposure experiment using a lower concentration of Kanto loam powder is required.
